# Ketamine infusion for patients receiving extracorporeal membrane oxygenation support: a case series

**DOI:** 10.12688/f1000research.6006.1

**Published:** 2015-01-16

**Authors:** Bethany Tellor, Nicole Shin, Thomas J. Graetz, Michael S. Avidan

**Affiliations:** 1 Pharmacy Department, Barnes-Jewish Hospital, St Louis, MO, USA; 2 Washington University School of Medicine, St Louis, MO, USA; 3 Department of Anesthesiology, Washington University School of Medicine, St Louis, MO, USA

**Keywords:** Ketamine, Extracorporeal membrane oxygenation, Intensive care unit, Critically-ill, Sedation

## Abstract

The use of ketamine infusion for sedation/analgesia in patients receiving extracorporeal membrane oxygenation (ECMO) therapy has not been described. The aims of this retrospective cohort study were to explore whether ketamine infusion for patients requiring ECMO therapy was associated with altered RASS scores, decreased concurrent sedative or opioid use, or with changes in vasopressor requirements.

All patients on ECMO who received ketamine infusions in addition to sedative and/or opioid infusions between December 2013 and October 2014 at Barnes-Jewish Hospital in St. Louis were retrospectively identified. Patient characteristics and process of care data were collected.

A total of 26 ECMO patients receiving ketamine infusion were identified. The median (inter quartile range [range]) age was 40 years (30-52 [25-66]) with 62% male. The median starting infusion rate of ketamine was 50 mg/hr (30-50 [6-150]) and it was continued for a median duration of 9 days (4-14 [0.2-21]). Prior to ketamine, 14/26 patients were receiving vasopressor infusions to maintain hemodynamic stability. Ketamine initiation was associated with a decrease in vasopressor requirement in 11/26  patients within two hours, and 0/26 required an increase (p<0.001). All patients were receiving sedative and/or opioid infusions at the time of ketamine initiation; 9/26 had a decrease in these infusions within two hours of ketamine initiation, and 1/26 had an increase (p=0.02; odds ratio for decrease to increase = 9; 95% CI, 1.14 to 71.04). The median (IQR[range]) RASS score 24 hours before ketamine initiation was -4 (-3 to -5, [0 to -5]) and after ketamine was -4 (-3 to -4 [-1 to -5]) ( *P* = 0.614).

Ketamine infusion can be used as an adjunctive sedative agent in patients receiving ECMO and may decrease concurrent sedative and/or opioid infusions without altering RASS scores. The hemodynamic effects of ketamine may provide the benefit of decreasing vasopressor requirements.

## Introduction

Continuous infusion of sedatives and opioids are commonly employed to alleviate pain, agitation, and anxiety in critically ill patients admitted to the intensive care unit ^1– 2^. Significant variability in sedation practices exist nationwide in the United States ^3^, and efforts have been made to investigate systematic approaches to sedation management in order to improve patient outcomes ^4^. As the adverse effects of over-sedation and prolonged sedation have become more apparent (e.g. longer duration of mechanical ventilation, longer ICU length of stay, increased mortality) ^5– 7^, the paradigm has shifted towards a “more is less” strategy ^8^. Keeping critically ill patients less sedated and more interactive is supported by recent clinical practice guidelines on pain, agitation, and delirium ^9^. However, severe agitation is common in critically ill patients and may have many underlying causes; agitation in such patients is associated with prolonged ventilator and ICU days as well as higher rates of self-extubation ^10^. It is therefore important to determine the optimum level of sedation for every individual critically ill patient. Patients requiring extracorporeal membrane oxygenation (ECMO) therapy typically require sedation during the acute phase of their illness or when they are agitated such that they are at risk for self-harm, such as displacement of ECMO tubing. However, patients receiving ECMO support are challenging to sedate, often require escalating doses of sedatives and opioids to maintain an appropriate, safe level of sedation ^11^. The challenges often relate to the dynamic alterations in pharmacokinetic (PK) and pharmacodynamic (PD) properties of commonly used sedatives and opioids in the setting of ECMO. Hemodilution, protein binding changes, changes in blood flow especially through the lungs, sequestration of medications by the ECMO circuit, binding of drugs to the oxygenator membrane, and organ function changes may impact the clinical effects of sedative and analgesic medications in this patient population ^10– 19^.

Ketamine is potentially a promising complementary sedative agent in the setting of ECMO in that it has hypnotic, analgesic, and amnesic properties owing to its activity at a variety of receptors. Ketamine is an antagonist of glutamate receptors as well as N-methyl-D-aspartate (NMDA) receptors and it has activity at all opioid receptors, though with different affinities and activities at those receptors. Ketamine also antagonizes nicotinic acetylcholine receptors noncompetitively and profoundly inhibits muscarinic receptors in addition to inhibiting L-type calcium channels ^20^. Ketamine’s lack of respiratory depression, bronchodilating properties, in addition to generally being well tolerated hemodynamically (despite some myocardial depressant properties) theoretically make it an attractive choice for sedation and analgesia ^21^. Recent clinical practice guidelines on pain, agitation, and delirium ^9^ mention the use of ketamine as a possible adjunct for non-neuropathic pain management and suggest that nonbenzodiazepine sedatives such as propofol and dexmedetomidine may be preferred over benzodiazepine sedation. They do not provide recommendations for ketamine use as a continuous infusion. To our knowledge, no studies have compared clinical outcomes in ICU patients sedated with ketamine infusion to other agents. Ketamine may provide an alternative or adjunct option in difficult to sedate patients. As critical drug shortages continue to impact the selection of available drugs (e.g. propofol shortage ^22^), it is important to review all relevant agents available for clinical use. The purpose of this study was to describe the use of ketamine infusion for patients receiving concurrent sedation/analgesia on ECMO support. The specific aims were to determine whether ketamine altered Richmond Agitation Sedation Scale (RASS) scores or decreased concurrent sedative, opioid, or vasopressor requirements.

## Methods

The Washington University School of Medicine Human Research Protection Office and the Protocol Review and Monitoring Committee approved this study (IRB # 201409142). This case series was conducted at Barnes-Jewish Hospital, a 1,250 bed urban teaching hospital affiliated with the Washington University School of Medicine in St. Louis, MO, between December 2013 and October 2014. All patients on ECMO support receiving ketamine infusion in addition to sedative and/or opioid infusions during the study period were retrospectively identified via an informatics query. There were no exclusion criteria. Patient characteristics, medical and surgical history, as well as process of care data were collected from electronic medical records. All ECMO patients are cared for in a single cardiothoracic intensive care unit. Doses and durations of all infusions of sedatives, opioids and vasopressor agents documented as administered during ketamine administration were collected. All available RASS scores charted 24 hours before and after ketamine initiation were collected on each patient. Statistical analysis was completed by using the SPSS software, version 18.0 (SPSS, Inc., Chicago, IL). Descriptive statistics were used to analyze the data collected. The median RASS scores as well as the median norepinephrine infusion rates (in patients who were receiving norepinephrine) before and after ketamine initiation were compared with a Wilcoxon signed-rank test. The McNemar test was used to assess whether or not a significant proportion of patients had a clinically meaningful increase or decrease in the dose of sedative, analgesic or vasopressor infusions two hours after ketamine initiation. Based on consensus of the investigators, the following minimum changes were arbitrarily pre-specified as clinically meaningful for sedative, analgesic and vasopressor infusions: propofol, 10 mcg/kg/min; midazolam, 1 mg/hour; dexmedetomidine, 0.2 mcg/kg/hour; fentanyl, 25 mcg/hour; norepinephrine 0.02 mcg/kg/min; and vasopressin 0.02 units/min.

## Results

**Ketamine infusion for sedation and analgesia data for patients receiving extracorporeal membrane oxygenation support**

Dataset 1_Patient demographics of patients receiving ketamine infusions while on extracorporeal membrane oxygenation therapy. Dataset 2_Continuous infusion details of patients receiving ketamine infusions while on extracorporeal membrane oxygenation therapy. Dataset 3_Vasopressor and sedative/opioid changes of patients receiving ketamine infusions while on extracorporeal membrane oxygenation therapy. Dataset 4_RASS scores before and after ketamine of patients receiving ketamine infusions while on extracorporeal membrane oxygenation therapy.

Click here to access the data: 10.7910/DVN/28617

A total of 26 ECMO patients receiving ketamine infusion were identified. The median (inter quartile range [range]) patient age was 40 years (30–52 [25–66]) with 62% male. The most frequent indication for ECMO support was respiratory failure associated with H1N1 influenza (46%). The median starting infusion rate of ketamine was 50 mg/hr (30–50 [6–150]) and it was continued for a median duration of 9 days (4–14 [0.2–21]). If the patient died in the hospital, that was considered the end of hospital stay. The median time from ECMO placement to ketamine initiation was 6 days (2–9 [0.9–22]). Nine patients (35%) died during their hospital stay. The most common causes of death were cardiopulmonary arrest and respiratory failure ( Table 1).

**Table 1.** Demographic data.

Characteristic	n = 26
Age, median (IQR, range), y	41 (30–52, 25–66)
Male sex	16 (62)
Body weight, median (IQR, range), kg	91 (72–111, 41–167)
Type of ECMO	
VV alone	14 (54)
VV and VA	7 (27)
VA alone	5 (19)
Comorbidities
Systolic heart failure	8 (31)
Hypertension	9 (35)
Atrial Fibrillation	7 (27)
Diabetes mellitus	6 (23)
Coronary artery disease	5 (19)
Hospital admitting diagnosis (not necessarily indication for ECMO support)	
H1N1	12 (46)
Cardiogenic shock	4 (15)
Acute respiratory distress syndrome	3 (12)
Decompensated heart failure	3 (12)
Other*	4 (15)
ICU length of stay, median (IQR, range), d	45.5 (25.7–70, 3.6–123.7)
Hospital length of stay, median (IQR, range), d Mortality during hospital stay ^£^	50 (26–69, 3.6–170) 9 (35)

Values are expressed as n (%) unless specified otherwise.

ECMO = extracorporeal membrane oxygenation; VV = veno-venous; VA = veno-arterial; IQR = interquartile range

*Other = leukemia, Stiffman syndrome, cystic fibrosis, pneumonia

^£^Cause of death documented in death summary note = cardiopulmonary arrest (2), respiratory failure (3), cardiogenic shock (2), acute respiratory distress syndrome (1), Hemophagocytic lymphohistiocytosis (1)

All patients were on a combination of one or more of the following agents: fentanyl, midazolam, propofol, and/or dexmedetomidine. At the time of ketamine initiation the median concurrent infusion rates of fentanyl (n=25), midazolam (n=19), propofol (n=10), and dexmedetomidine (n=9) were 200 mcg/hr (150–450 [50–900]), 7 mg/hr (4–9 [1–15]), 40 mcg/kg/min (23–48 [10–100]), and 1.2 mcg/kg/hr (1–1.4 [0.6–1.5]), respectively. The median starting infusion rate of ketamine was 50 mg/hr (30–50 [6–150]) and continued for a median duration of 9 days (4–14 [0.2–21]). Of note, 6 patients were receiving concurrent neuromuscular blocking agents during ketamine infusion ( Table 2).

**Table 2.** Continuous infusions.

Continuous Infusion	n = 26
Ketamine, n (%)	26 (100)
Duration of infusion, d	9 (4–14, 0.2–21)
Initial rate, mg/hr	50 (30–50, 6–150)
Maximum rate, mg/hr	150 (65–250, 10–300)
Minimum rate, mg/hr	50 (30–50, 6–150)
Fentanyl, n (%)	25 (96)
Rate when ketamine initiated, mcg/min	200 (150–400, 50–900)
Maximum rate, mcg/hr	500 (200–600, 100–900)
Minimum rate, mcg/hr	200 (100–350, 50–600)
Midazolam, n (%)	19 (73)
Rate when ketamine initiated, mg/hr	7 (4–9, 1–15)
Maximum rate, mg/hr	10 (8–12, 2–20)
Minimum rate, mg/hr	2 (2–6, 1–10)
Propofol, n (%)	10 (38)
Rate when ketamine initiated, mcg/kg/min	40 (23–48, 10–100)
Maximum rate, mcg/kg/min	30 (20–50, 15–100)
Minimum rate, mcg/kg/min	20 (11–30, 0–100)
Dexedetomidine, n (%)	9 (35)
Rate when ketamine initiated, mcg/kg/hr	1.2 (1–1.4, 0.6–1.5)
Maximum rate, mcg/kg/hr	1(0.7–1.5, 0.6–1.5)
Minimum rate, mcg/kg/hr	0.5 (0.07–1, 0–1.5)
Neuromuscular blocking agents, n (%)	6 (23)

Values are median (IQR, range), unless specified otherwise

IQR = interquartile range.

Prior to ketamine, 14/26 patients were receiving vasopressor infusions to maintain hemodynamic stability. Ketamine initiation was associated with a clinically meaningful decrease in vasopressor requirement in 11/26 patients within two hours, and 0/26 required an increase in this time period (p<0.001). In the 14 patients on norepinephrine infusions, the median (IQR [range]) dose before ketamine initiation was 0.1 mcg/kg/min (0.04–0.19 [0.02–0.26]) and two hours after ketamine initiation was 0.06 mcg/kg/min (0.01–0.11 [0–0.26]) (P=0.003). All patients were receiving sedative and/or opioid infusions at the time of ketamine initiation; 9/26 had a clinically meaningful decrease in these infusions within two hours of ketamine initiation, and 1/26 had an increase (p=0.02; odds ratio for decrease to increase = 9; 95% CI, 1.14 to 71.04) ( Table 3). The median RASS score 24 hours before ketamine initiation was -4 (-3 to -5, [0 to -5]) and after ketamine was -4 (-3 to -4 [-1 to -5]) ( *P*=0.614).

**Table 3.** Concurrent vasopressor, sedative/analgesic requirements.

Medications	Any meaningful* dose decrease within 2 hours of ketamine initiation	Any meaningful* dose increase within 2 hours of ketamine initiation	P value
Vasopressors (n = 14)	11	0	<0.001
Concurrent Analgesia/ Sedation (n = 26)	9	1	0.02

*Based on consensus of the investigators, the following minimum changes were arbitrarily pre-specified as clinically meaningful for sedative, analgesic and vasopressor infusions: propofol, 10 mcg/kg/min; midazolam, 1 mg/hour; dexmedetomidine, 0.2 mcg/kg/hour; fentanyl, 25 mcg/hour; norepinephrine 0.02 mcg/kg/min; and vasopressin 0.02 units/min.

## Discussion

ECMO has repeatedly shown to complicate sedation management and optimization in the ICU as the PK/PD of sedatives and opioids become altered ^11, 12, 14– 18^. Lorazepam, midazolam, diazepam, propofol, and morphine have all been shown to be sequestered by ECMO circuits to some extent ^11, 17, 18^, posing a challenge when faced with trying to provide appropriate sedation/analgesia. In an *ex vivo* study, lipophilic medications such as fentanyl and midazolam have been shown to sequester in the ECMO circuit resulting in significant loss, while morphine remained relatively stable ^18^. A similar finding was shown by Shekar and collegaues ^11^ who assessed sedation requirements in 30 patients receiving ECMO support. Significant increases in dosing requirements of midazolam and morphine were noted, with venovenous ECMO patients receiving higher sedative doses overall than patients on venoarterial ECMO. The effect of ECMO on the PK/PD and other drug properties of ketamine in patients on ECMO has not been studied.

Ketamine causes dissociation of the thalamus from the limbic cortex, resulting in patients resembling a cataleptic state. Patients may be unable to respond to sensory stimulation, may have nystagmus, and are able to conserve laryngeal and corneal reflexes ^21^. Ketamine has been shown to decrease opioid consumption in surgical patients ^23, 24^, and is typically used as a one-time administration for analgesia, anesthesia induction, or procedural sedation. In addition to these indications, in some of the ICUs at our institution ketamine infusion has been added for patients who have been difficult to sedate to a target RASS. The use of ketamine infusion has particularly been adopted at our institution for patients requiring ECMO therapy, as these patients have anecdotally been difficult to sedate optimally. However, the safety and efficacy of ketamine for these patients has not been established.

The favorable hemodynamic and PK/PD profile that ketamine provides in patients not requiring ECMO therapy makes ketamine theoretically an attractive sedative agent generally for use in the ICU. The onset of action after IV administration occurs within 30 seconds, with a maximum effect in about 1 minute. The distribution half-life is 5–10 minutes while the elimination half-life is 2–3 hours, as ketamine undergoes extensive hepatic uptake and is primarily excreted in the urine ^21, 25^. There are no dosing adjustments provided by the manufacturer in the presence of hepatic or renal dysfunction, however the optimal dose and duration of ketamine infusion for sedation remains unknown. Interestingly, in recent years neuroprotection, immune modulating, and antidepressant properties have also been suggested for ketamine ^26– 28^.

Ketamine’s lack of respiratory depression, bronchodilation properties and ability to increase blood pressure, heart rate, and cardiac output (although it does act as a myocardial depressant) further add to the attractive profile of this agent ^21, 29^. Interestingly, a decrease in vasopressor requirements with ketamine initiation was observed in many of the patients in this series. Although charted RASS scores before and during ketamine infusion did not change, a decrease in sedatives and/or opioids within two hours of ketamine initiation was more common than an increase in sedatives and/or opioids.

This study has several limitations. The small sample size, lack of blinding and retrospective design limit our ability to make causal inferences. Although the majority of patients receiving vasopressors had a meaningful decrease in their requirements, we cannot exclude the fact that a decrease in vasopressors may have occurred unrelated to ketamine infusion. The observational design also limits our ability to determine why certain drugs were chosen concurrently or why one was decreased prior to another. Although RASS values were collected, we were unable to determine the specified RASS goal for each patient. A RASS goal of 0 to -2 is targeted in most ICU patients, however 6 patients in this series were receiving neuromuscular blocking agents, rendering RASS values uninterpretable, while other patients received escalating doses of sedatives/opioids despite RASS values < -2. Furthermore, the use of a RASS score to monitor and guide sedation with ketamine has not been validated.

To our knowledge, this is the first study evaluating the use of ketamine infusion in patients on ECMO support. This study demonstrated that ketamine infusion can be used as an adjunctive agent in patients receiving ECMO with possible sedative/opioid sparing effects. The cardiovascular properties may provide the hemodynamic benefit of reducing vasopressor requirements. The lack of understanding related to optimizing pharmacotherapy in patients on ECMO has led to ongoing research, aimed at improving patient care ^13, 19, 30^. All appropriate drugs should be considered in the absence of clear standards or guidelines for analgesia/sedation in patients receiving ECMO support. More rigorous study is warranted to further investigate the suitability of ketamine for sedation in patients receiving ECMO support.

## Data availability

Dataverse: Dataset 1. Ketamine infusion for sedation and analgesia data for patients receiving extracorporeal membrane oxygenation support, doi: 10.7910/DVN/28617 ^31^

## References

[1] Payen JF Chanques G Mantz J: Current practices in sedation and analgesia for mechanically ventilated critically ill patients: a prospective multicenter patient-based study. *Anesthesiology.* 2007;106(4):687–95. 10.1097/01.anes.0000264747.09017.da 17413906

[2] Patel SB Kress JP: Sedation and analgesia in the mechanically ventilated patient. *Am J Respir Crit Care Med.* 2012;185(5):486–497. 10.1164/rccm.201102-0273CI 22016443

[3] Rhoney DH Murry KR: National survey of the use of sedating drugs, neuromuscular blocking agents, and reversal agents in the intensive care unit. *J Intensive Care Med.* 2003;18(3):139–45. 10.1177/0885066603251200 14984632

[4] Jackson DL Proudfoot CW Cann KF: A systematic review of the impact of sedation practice in the ICU on resource use, costs and patient safety. *Crit Care.* 2010;14(2):R59. 10.1186/cc8956 20380720 PMC2887180

[5] Shehabi Y Bellomo R Reade MC: Sedation Practice in Intensive Care Evaluation (SPICE) Study Investigators; ANZICS Clinical Trials Group: Early intensive care sedation predicts long-term mortality inventilated critically ill patients. *Am J Respir Crit Care Med.* 2012;186(5):724–731. 10.1164/rccm.201203-0522OC 22859526

[6] Shehabi Y Chan L Kadiman S: Sedation Practice in Intensive Care Evaluation (SPICE) Study Group investigators: Sedation depth and long-term mortality in mechanically ventilated critically ill adults: a prospective longitudinal multicentre cohort study. *Intensive Care Med.* 2013;39(5):910–918. 10.1007/s00134-013-2830-2 23344834 PMC3625407

[7] Kollef MH Levy NT Ahrens TS: The use of continuous i.v. sedation is associated with prolongation of mechanical ventilation. *Chest.* 1998;114(2):541–8. 10.1378/chest.114.2.541 9726743

[8] Peitz GJ Balas MC Olsen KM: Top 10 myths regarding sedation and delirium in the ICU. *Crit Care Med.* 2013;41(9 Suppl 1):S46–56. 10.1097/CCM.0b013e3182a168f5 23989095

[9] Barr J Fraser GL Puntillo K: Clinical practice guidelines for the management of pain, agitation, and delirium in adult patients in the intensive care unit. *Crit Care Med.* 2013;41(1):263–306. 10.1097/CCM.0b013e3182783b72 23269131

[10] Woods JC Mion LC Connor JT: Severe agitation among ventilated medical intensive care unit patients: frequency, characteristics and outcomes. *Intensive Care Med.* 2004;30(6):1066–1072. 10.1007/s00134-004-2193-9 14966671

[11] Shekar K Roberts JA Mullany DV: Increased sedation requirements in patients receiving extracorporeal membrane oxygenation for respiratory and cardio-respiratory failure. *Anaesth Intensive Care.* 2012;40(4):648–655. 22813493 10.1177/0310057X1204000411

[12] Shekar K Fraser JF Smith MT: Pharmacokinetic changes in patients receiving extracorporeal membrane oxygenation. *J Crit Care.* 2012;27(6):741.e9–741.e18. 10.1016/j.jcrc.2012.02.013 22520488

[13] Shekar K Roberts JA Smith MT: The ECMO PK Project: an incremental research approach to advance understanding of the pharmacokinetic alterations and improve patient outcomes during extracorporeal membrane oxygenation. *BMC Anesthesiol.* 2013;13:7. 10.1186/1471-2253-13-7 23517311 PMC3643838

[14] Ahsman MJ Hanekamp M Wildschut ED: Population pharmacokinetics of midazolam and its metabolites during venoarterial extracorporeal membrane oxygenation in neonates. *Clin Pharmacokinet.* 2010;49(6):407–419. 10.2165/11319970-000000000-00000 20481651

[15] Dagan O Klein J Bohn D: Effects of extracorporeal membrane oxygenation on morphine pharmacokinetics in infants. *Crit Care Med.* 1994;22(7):1099–1101. 10.1097/00003246-199407000-00008 8026197

[16] Mulla H Lawson G Peek GJ: Plasma concentrations of midazolam in neonates receiving extracorporeal membrane oxygenation. *ASAIO J.* 2003;49(1):41–47. 12558306 10.1097/00002480-200301000-00007

[17] Mulla H Lawson G von Anrep C: *In vitro* evaluation of sedative drug losses during extracorporeal membrane oxygenation. *Perfusion.* 2000;15(1):21–26. 10.1177/026765910001500104 10676864

[18] Shekar K Roberts JA Mcdonald CI: Sequestration of drugs in the circuit may lead to therapeutic failure during extracorporeal membrane oxygenation. *Crit Care.* 2012;16(5):R194. 10.1186/cc11679 23068416 PMC3682296

[19] Shekar K Roberts JA Welch S: ASAP ECMO: Antibiotic, Sedative and Analgesic Pharmocokinetics during Extracorporeal Membrane Oxygenation: a multi-centre study to optimize during therapy during ECMO. *BMC Anesthesiol.* 2012;12:29 10.1186/1471-2253-12-29 23190792 PMC3543712

[20] Sinner B Graf BM: Ketamine. *Handbook of experimental pharmacology.* 2008;182:313–33. 10.1007/978-3-540-74806-9_15 18175098

[21] Parashchanka A Schelfout S Coppens M: Role of novel drugs in sedation outside the operating room: dexmedetomidine, ketamine and remifentanil. *Curr Opinion Anesthesiol.* 2014;27(4):442–7. 10.1097/ACO.0000000000000086 24762954

[22] Jensen V Rappaport BA: The reality of drug shortages--the case of the injectable agent propofol. *N Engl J Med.* 2010;363(9):806–07. 10.1056/NEJMp1005849 20554977

[23] De Pinto M Jelacic J Edwards WT: Very-low-dose ketamine for the management of pain and sedation in the ICU. *J Opioid Manag.* 2008;4(1):54–6. 10.1016/j.acpain.2008.05.023 18444449

[24] Zakine J Samarcq D Lorne E: Postoperative ketamine administration decreases morphine consumption in major abdominal surgery: a prospective, randomized, double-blind, controlled study. *Anesth Analg.* 2008;106(6):1856–61. 10.1213/ane.0b013e3181732776 18499623

[25] Ketamine hydrochloride injection [prescribing information]. Lake Forest, IL: Hospira Inc; January2013 Reference Source

[26] Hudetz JA Pagel PS: Neuroprotection by ketamine: a review of the experimental and clinical evidence. *J Cardiothorac Vasc Anesth.* 2010;24(1):131–42. 10.1053/j.jvca.2009.05.008 19640746

[27] Murrough JW: Ketamine as a novel antidepressant: from synapse to behavior. *Clin Pharmacol Ther.* 2012;91(2):303–9. 22205190 10.1038/clpt.2011.244 PMC3673880

[28] Mathews DC Zarate CA: Current status of ketamine and related compounds for depression. *J Clin Psychiatry.* 2013;74(5):516–517. 10.4088/JCP.13ac08382 23759454 PMC3785314

[29] Miller AC Jamin CT Elamin EM: Continuous intravenous infusion of ketamine for maintenance sedation. *Minerva Anestesiol.* 2011;77(8):812–820. 21730929

[30] Columbia University, Ketamine and Extracorporeal Membrane Oxygenation (ECMO).In: ClinicalTrials.gov [cited 2014 Dec 18]. Reference Source

[31] Tellor B Shin N Graetz TJ: Ketamine infusion for sedation and analgesia data for patients receiving extracorporeal membrane oxygenation support. *Dataverse.* 2014 Data Source 10.12688/f1000research.6006.1 PMC4536611 26309726

