# Supplementary material for: Ketamine infusion for patients receiving extracorporeal membrane oxygenation support: a case series
Source: F1000Res. 2015 Jan 16;4:16. [Version 1] doi: 10.12688/f1000research.6006.1 (PMC4536611; doi:10.12688/f1000research.6006.1)
Supplement: Peer review history

# Peer review: Referee response for version 1

**DOI:** 10.5256/f1000research.6427.r7631

**Published:** 2015-03-05

**Authors:** Anthony Absalom, Marko Sahinovic

**Affiliations:** 1 Department of Anaesthesiology, University Medical Center Groningen, Groningen, Netherlands; 2 Department of Anaesthesiology, University Medical Center Groningen, Groningen, GZ, 9733 HD, Netherlands

**Summary:**

The authors should be congratulated on an excellent article reporting an analysis of routinely collected clinical data. They have addressed a clinically relevant subject and with an important research question and a plausible and intuitive underlying hypothesis. Although retrospective, the study methodology is good and the statistical analysis is appropriate. The results are clear, although the manner of presentation could be improved. The discussion is informative and educational. The study limitations are appropriately recognised and discussed, and suggestions for future investigation are made. The conclusions are well supported by the data.

**Abstract**

Clear and concise. It describes the background, results and the conclusions accurately.

**Introduction**

The introduction provides a clear and concise summary of the clinical background and relevance of the study. The proposed hypothesis is clinically very relevant and plausible. The specific aims of the study are stated clearly.

**Methods**

The description of the methods is completely clear. Appropriate methodology was applied. To assess the influence of ketamine on sedative and vasopressor requirements, the authors arbitrarily chose, by consensus, definitions of “ *clinically meaningful minimal changes in medication after the start of ketamine infusion*”.  This is a reasonable approach. The statistical analysis is clearly described and is appropriate. 

**Results**

We have two comments about the presentation of the results. The first is that table 2 is somewhat confusing. It is not clear what the “maximum” and “minimum” infusion rates refer to. We suggest that the authors clearly state the time period over which these maximum and minimum infusion rates were determined. If these rates are not the rates after starting ketamine, then we suggest that the authors present the median (IQR, range) infusion rates during some (arbitrary) time period after starting ketamine.

Our second comment about the results concerns the consistency of units used in presentation of sedative and vasopressor infusion rates. In the methods the authors state arbitrarily chosen definitions of clinically meaningful changes in medication. These thresholds are defined in mcg/kg/min units for each of the drugs. In the text and in table 2 of the results, the infusion rates of fentanyl and midazolam are reported in different units (mcg/min). Thereafter the authors only report the numbers of patients in which there was a clinically relevant change.  To more easily give the reader an idea of the magnitude of the relative changes in infusion rate of fentanyl and midazolam, we suggest that for these drugs the before and ketamine infusion rates are also presented in mcg/kg/min.

**Discussion**

Discussion is very well written and informative. The study limitations are well recognised. The conclusions are supported by the data.

**References**

Appropriate use of references

We have read this submission. We believe that we have an appropriate level of expertise to confirm that it is of an acceptable scientific standard.

# Peer review: Referee response for version 1

**DOI:** 10.5256/f1000research.6427.r7693

**Published:** 2015-02-18

**Authors:** Kevin Thornton

**Affiliations:** 1 Department of Anesthesia and Perioperative Care, University of California, San Francisco, San Francisco, CA, USA

This article is a retrospective case series reviewing the use of ketamine as an adjunctive sedative agent for patients requiring ECMO support due to cardiopulmonary failure. The authors reviewed patient characteristics including concurrent sedative, analgesic, and vasopressor infusion requirements as well as RASS scores both before and after initiation of ketamine infusion. The authors defined what they considered to be a 'clinically significant' changes in the doses of several agents including fentanyl, midazolam, propofol, and norepinephrine. While the majority of patients had a 'clinically significant' decrease in the doses of both vasopressors and other sedative/analgesic agents, these changes were still quite small. The authors rightly note that they are not able to make a causal associations given the retrospective nature of their data, but their inferences are sound based on pharmacologic and physiologic principles. This case series demonstrates that ketamine appears to be an adjunctive agent that can safely be used in patients on ECMO and may have the benefit of decreasing the requirements for other sedatives and vasopressors, as well. This article is well-written and this topic warrants further investigation in a prospective manner.

I have read this submission. I believe that I have an appropriate level of expertise to confirm that it is of an acceptable scientific standard.
